# Correlates of Transitions in Tobacco Product Use by U.S. Adult Tobacco Users between 2013–2014 and 2014–2015: Findings from the PATH Study Wave 1 and Wave 2

**DOI:** 10.3390/ijerph15112556

**Published:** 2018-11-14

**Authors:** Karin A. Kasza, Blair Coleman, Eva Sharma, Kevin P. Conway, K. Michael Cummings, Maciej L. Goniewicz, Raymond S. Niaura, Elizabeth Y. Lambert, Liane M. Schneller, Shari P. Feirman, Elisabeth A. Donaldson, Yu-Ching Cheng, Iilun Murphy, Jennifer L. Pearson, Dennis R. Trinidad, Maansi Bansal-Travers, Tara Elton-Marshall, Daniel A. Gundersen, Cassandra A. Stanton, David B. Abrams, Geoffrey T. Fong, Nicolette Borek, Wilson M. Compton, Andrew J. Hyland

**Affiliations:** 1 Department of Health Behavior, Roswell Park Comprehensive Cancer Center, Buffalo, NY 14263, USA; Maciej.Goniewicz@RoswellPark.org (M.L.G.); Liane.Schneller@RoswellPark.org (L.M.S.); Maansi.Travers@RoswellPark.org (M.B.-T.); Andrew.Hyland@RoswellPark.org (A.J.H.); 2 Office of Science, Center for Tobacco Products, U.S. Food and Drug Administration, Silver Spring, MD 20993, USA; Blair.Coleman@fda.hhs.gov (B.C.); Shari.Feirman@fda.hhs.gov (S.P.F.); Elisabeth.Sherman@fda.hhs.gov (E.A.D.); Yu-Ching.Cheng@fda.hhs.gov (Y.C.-C.); Iilun.Murphy@fda.hhs.gov (I.M.); Nicolette.Borek@fda.hhs.gov (N.B.); 3 Westat, Rockville, MD 20850, USA; EvaSharma@westat.com (E.S.); CassandraStanton@westat.com (C.A.S.); 4 National Institute on Drug Abuse, National Institutes of Health, Bethesda, MD 20892, USA; kconway@rti.org (K.P.C.); elambert@nida.nih.gov (E.Y.L.); wcompton@nida.nih.gov (W.M.C.); 5 Department of Psychiatry and Behavioral Sciences, Medical University of South Carolina, Charleston, SC 29425, USA; cummingk@musc.edu; 6 The Schroeder Institute for Tobacco Research and Policy Studies, Truth Initiative, Washington, DC 20001, USA; niaura@nyu.edu (R.S.N.); jennipearson@unr.edu (J.L.P.); david.b.abrams@nyu.edu (D.B.A.); 7 Department of Family Medicine and Public Health, University of California, La Jolla, CA 92093, USA; dtrinidad@ucsd.edu; 8 Institute for Mental Health Policy Research, Centre for Addiction and Mental Health, London, ON M5T 1R8, Canada; Tara.EltonMarshall@camh.ca; 9 Dalla Lana School of Public Health, University of Toronto, Toronto, ON M5S 1A1, Canada; 10 Department of Epidemiology and Biostatistics, Schulich School of Medicine and Dentistry, Western University, London, ON N6A 3K7, Canada; 11 Ontario Tobacco Research Unit, Toronto, ON M5S 2S1, Canada; 12 Department of Family Medicine and Community Health, Robert Wood Johnson Medical School, Rutgers, Somerset, NJ 08873, USA; DanielA_Gundersen@dfci.harvard.edu; 13 Survey and Data Management Core, Dana-Farber Cancer Institute, Boston, MA 02215, USA; 14 School of Public Health and Health Systems, University of Waterloo, Waterloo, ON N2L 3G1, Canada; geoffrey.fong@uwaterloo.ca; 15 Department of Psychology, University of Waterloo, Waterloo, ON N2L 3G1, Canada; 16 Ontario Institute for Cancer Research, Toronto, ON M5G 0A3, Canada

**Keywords:** tobacco, transition, population, longitudinal, epidemiology, cigarettes, electronic nicotine delivery systems (ENDS), correlate, demographic, dependence

## Abstract

More than half of adult tobacco users in the United States (U.S.) transitioned in tobacco product use between 2013–2014 and 2014–2015. We examine how characteristics of adult tobacco users in the U.S. relate to transitions in tobacco product use. Population Assessment of Tobacco and Health (PATH) Study data were analyzed from 12,862 adult current tobacco users who participated in Wave 1 (W1, 2013–2014) and Wave 2 (W2, 2014–2015). Three types of transitions were examined—(1) adding tobacco product(s); (2) switching to non-cigarette tobacco product(s); and (3) discontinuing all tobacco use—among those currently using: (1) any tobacco product; (2) cigarettes only (i.e., exclusive cigarette); and (3) cigarettes plus another tobacco product(s) (i.e., poly-cigarette). Multinomial logistic regression analyses determined relative risk of type of transition versus no transition as a function of demographic and tobacco use characteristics. Transitions in tobacco product use among adult tobacco users were common overall, but varied among different demographic groups, including by age, sex, sexual orientation, race/ethnicity, educational attainment, and poverty level. Further, cigarette smokers with higher dependence scores were more likely to add product(s) and less likely to discontinue tobacco use compared to those with low dependence scores. That high nicotine dependence is a barrier to discontinuing tobacco use adds evidence to support policy to lower nicotine content of cigarettes and to evaluate new products for their potential to reduce cigarette use.

## 1. Introduction

As the tobacco product landscape in the United States (U.S.) expands [1,2,3] and prevalence of use of non-cigarette tobacco products increases [4,5,6], longitudinal data reveal that transitions in use of tobacco products are common among adult tobacco users in the U.S. That is, data from the Population Assessment of Tobacco and Health (PATH) Study indicate that over half of adult tobacco users transitioned in product use or combination of products used between 2013–2014 and 2014–2015, though cigarettes still remain the predominate type of tobacco product used by adults [7]. Understanding the population-level impact of the changing tobacco landscape requires determining both whether and how cigarette smokers and other tobacco product users transition in their product use over time, as well as whether transitions differ by demographic or tobacco use characteristics, such as frequency of use, number of products used, and indicators of nicotine dependence. 

Prior research shows that age [7,8] and frequency of product use [9] are each associated with transitions in tobacco use, with younger adults and non-daily cigarette smokers being more likely to transition among tobacco products than their older adult and daily cigarette smoker counterparts. However, prior studies analyzing correlates of transitions among types of tobacco products have not considered the full range of new and emerging tobacco products available today, nor have they addressed a wider range of demographic characteristics that have historically been associated with tobacco use, such as race/ethnicity, sexual orientation, and socioeconomic status [10,11,12,13,14,15]. Additionally, a contemporary examination of how nicotine dependence relates to transitions in tobacco product use within the changing tobacco marketplace can help inform the U.S. Food and Drug Administration’s plan to reduce the addictiveness of cigarettes by limiting their nicotine content [16].

This paper characterizes the demographic correlates (i.e., age, sex, sexual orientation, race/ethnicity, educational attainment, and poverty level) and tobacco use correlates (i.e., total number of products used, nicotine dependence, and type and frequency of product used) of transitions in tobacco product use in the U.S. over a one-year period (i.e., 2013–2014 to 2014–2015). Using Wave 1 (W1) and Wave 2 (W2) data from the PATH Study, we report relative likelihood of various types of transitions as a function of demographic and tobacco use correlates among all adult current tobacco users at W1, among adult current cigarette smokers who were not current users of other tobacco products at W1, and among adult current cigarette smokers who were also current users of at least one other type of tobacco product at W1.

## 2. Materials and Methods

### 2.1. Study Population

The PATH Study is an ongoing, nationally-representative, longitudinal cohort study of tobacco use and health among adults and youths in the U.S. [17]. W1 data collection was conducted from 12 September 2013 through 14 December 2014 and W2 data collection was conducted from 23 October 2014 through 30 October 2015. The PATH Study recruitment employed a stratified address-based, area-probability sampling design at W1 that oversampled adult tobacco users, young adults (18 to 24 years), and African Americans. An in-person screener was used at W1 to select adults and youths from households for participation. At W1, the weighted response rate for the household screener was 54.0%. Among households that were screened, the overall weighted response rate for the adult interview was 74.0% at W1 and 83.2% at W2. Audio computer-assisted self-interviews (ACASI) administered in English or Spanish were used to collect self-report information on tobacco-use patterns and associated health behaviors. All adult participants provided informed consent.

Data from 12,862 adult tobacco users (18+ years of age in W1) who participated in both W1 and W2 were used for this analysis. The weighted estimates presented here are representative of the non-institutionalized, civilian U.S. adult population through use of the probability sample and application of population and replicate weights that adjust for the complex study design characteristics (e.g., oversampling at W1) and nonresponse at W1 and W2. Nonresponse bias analyses for W1 and W2 are available at http://doi.org/10.3886/ICPSR36231 [18]. Further details regarding the PATH Study design and methods are published elsewhere [17]. Details on survey interview procedures, questionnaires, sampling, weighting, and information on accessing the data are available at http://www.icpsr.umich.edu/icpsrweb/NAHDAP/series/00606 [19]. The study was conducted by Westat and approved by the Westat institutional review board (IRB Organization number IORG0000410, IRB number 00000695).

### 2.2. Measures

#### 2.2.1. Types of Tobacco Products 

Participants were provided with brief descriptions and pictures of 10 types of tobacco products (except cigarettes) and were asked about their use of the products at each wave, which were grouped for analysis into six types: cigarettes, ENDS (e-cigarettes at W1, and e-cigarettes, e-cigars, e-pipes, and e-hookah at W2), cigars (i.e., traditional cigars, cigarillos, filtered cigars), pipe tobacco, hookah, and smokeless tobacco (i.e., loose snus, moist snuff, dip, spit, chewing tobacco, snus pouches, dissolvable tobacco). At W2, non-cigarette tobacco product types were additionally grouped into combustible products (i.e., cigars, pipe tobacco, and hookah) and non-combustible products (i.e., ENDS and smokeless tobacco). 

#### 2.2.2. Current Use of Tobacco Products 

At each wave, and for each type of tobacco product, participants were asked whether they now use the product every day, some days, or not at all. For hookah, participants were additionally asked whether they now use usually weekly or usually monthly. Current use at each wave was defined for cigarettes as having smoked at least 100 cigarettes in their lifetime and now smoking every day or somedays; for hookah as now smoking every day or somedays (including those who responded some days, usually weekly, or usually monthly; no threshold for units used); and for all other tobacco products as now smoking/using every day or somedays (no threshold for units used). For each tobacco product, a three-level current use variable was created to indicate daily use, someday use, or no use based on the criteria described above. The total number of products currently used was also calculated (complete data required).

#### 2.2.3. Tobacco Product User Groups

Tobacco product user groups were defined as follows: *Any tobacco*, defined as current use of any tobacco product(s); *Non-cigarette tobacco*, defined as current use of at least one tobacco product and no current use of cigarettes; *Exclusive cigarettes*, defined as current use of cigarettes and no current use of any other tobacco product; *Poly-cigarettes*, defined as current use of cigarettes and current use of at least one other tobacco product; *Cigarettes plus ENDS*, defined as current use of cigarettes and current use of ENDS (may also currently use any other tobacco product); *Cigarettes plus combustible*, defined as current use of cigarettes and current use of at least one other combustible tobacco product (may also currently use any non-combustible tobacco product); *Cigarettes plus non-combustible*, defined as current use of cigarettes and current use of at least one non-combustible tobacco product and no current use of any combustible tobacco product; *No cigarettes and no ENDS*, defined as no current use of cigarettes and no current use of ENDS (may currently use any other tobacco product); *No tobacco*, defined as no current use of any tobacco product. Some of these user groups overlap with each other to allow for the assessment of variations in user groups and transitions among them.

#### 2.2.4. Nicotine Dependence 

Sixteen items assessing symptoms of nicotine dependence were included in the PATH Study. Responses to these items were combined and scaled to produce an overall nicotine dependence score as described and validated by Strong and colleagues [20]. 

#### 2.2.5. Transitions in Tobacco Product Use 

Transitions in tobacco product use were determined among each of the following three tobacco user groups at W1 as defined above: *any tobacco users*, *exclusive cigarette smokers*, and *poly-cigarette smokers*. Among W1 *any tobacco* users (N = 12,862), transitions in tobacco product use between waves were determined using three mutually exclusive transition groups: (1) no transition, defined as current use of the exact same tobacco product(s) at W1 and W2; (2) transition among tobacco product(s), defined as any difference in current use of tobacco product(s) at W1 and W2 (i.e., any transition in type of product(s) used, which could result in an increase, decrease, or no change in number of products used) except for the case of no current tobacco use at W2; and (3) discontinue tobacco use, defined as no current tobacco use at W2. 

Among W1 *exclusive cigarette* smokers, transitions in tobacco product use between waves were determined with two sets of analyses: first, transitions were determined only with respect to ENDS using four mutually exclusive transition groups (N = 4893): (1) no transition, defined as cigarette use at W2 and no ENDS use at W2 (may use any other tobacco product at W2); (2) transition to cigarettes plus ENDS use; (3) switch to ENDS use, defined as ENDS use at W2 and no cigarette use at W2 (may use any other tobacco product at W2); and (4) transition to no cigarette use and no ENDS use at W2. Second, transitions in tobacco product use among *exclusive cigarette* smokers at W1 were determined with respect to all tobacco products using five mutually exclusive transition groups (N = 4846): (1) no transition, defined as exclusive cigarette use at W2; (2) transition to cigarettes plus combustible use; (3) transition to cigarettes plus non-combustible use; (4) switch to non-cigarette tobacco use; and (5) discontinue tobacco use. 

Among W1 *poly-cigarette* smokers (N = 3946), transitions in tobacco product use between waves were determined using four mutually exclusive transition groups: (1) no transition, defined as poly-cigarette use at W2 (which may reflect the same or different combinations of products used at W2 as were used at W1); (2) transition to exclusive cigarette use; (3) transition to non-cigarette tobacco use; and (4) discontinue tobacco use. 

#### 2.2.6. Demographic Characteristics 

At W1, participants were categorized according to age group (i.e., 18–24, 25–39, 40–54, 55+), sex (male, female), sexual orientation (i.e., heterosexual/straight, gay/lesbian/bisexual/something else), race/ethnicity (i.e., non-Hispanic white, non-Hispanic black or African American, non-Hispanic other, Hispanic), educational attainment (i.e., less than high school or GED, high school graduate, some college (no degree) or Associates degree, Bachelor’s degree or more), and household poverty level (i.e., below poverty level, at or near poverty level, at or above twice the poverty level, not reported). Missing data on race, ethnicity and education were imputed as described in the PATH Study Restricted Use Files User Guide (available at http://www.icpsr.umich.edu/icpsrweb/ NAHDAP/series/00606) [19]. 

### 2.3. Statistical Analysis 

Rates of transitions in tobacco product use between waves were determined among (1) *any tobacco* users at W1; (2) *exclusive cigarette* smokers at W1; and (3) *poly-cigarette* smokers at W1. For each of these three user groups at W1, transition rates were also stratified by demographic characteristics (i.e., age group, sex, sexual orientation, race/ethnicity, educational attainment, and household poverty level), and tobacco use variables assessed at W1 (i.e., total number of products currently used (not relevant for exclusive cigarette smokers), nicotine dependence (stratified into quartiles), and type and frequency of products currently used (i.e., no use, someday use, daily use)). These stratification variables are hereafter referred to as “correlates”.

Multinomial logistic regression analyses were used to determine the relative risk of being categorized into each transition group versus the “no transition” group as a function of correlates, using two models: Model 1 was adjusted for demographic variables only, and Model 2 was adjusted for all correlates (Model 1 consisted of separate analyses such that the analysis for each correlate was adjusted for demographic correlates only; Model 2 consisted of a single model that included all correlates at once). Analyses were conducted with Stata 14 software [21]. Estimates were weighted to represent the U.S. adult population and variances were estimated using the balanced repeated replication (BRR) method [22] with Fay’s adjustment set to 0.3 to increase estimate stability [23]. The logit-transformation method was used to calculate standard errors for the proportions. Estimates based on a sample size of less than 50 or with a relative standard error of greater than 30% are flagged in the tables.

## 3. Results

### 3.1. Any Tobacco User at W1

Table 1 shows rates and relative likelihood of transitions in tobacco product use among those using *any tobacco* at W1, stratified by correlates, and relative risk ratios (RRR) indicate the relative probabilities of being in each transition group versus the “no transition” group as a function of correlates assessed at W1. Overall, 47.9% of tobacco users did not transition in use of tobacco product(s), 37.6% transitioned among tobacco product(s), and 14.5% discontinued tobacco use.

#### 3.1.1. Correlates of Transitions among Tobacco Product(s) 

Young adults (i.e., those aged 18–24 years) were about five times more likely to transition among tobacco product(s) versus not transition than were adults aged 55+ years (Table 1, Model 1, RRR = 0.2, 95% CI = 0.2–0.2), and were more than twice as likely to transition after adjustment for tobacco use correlates (Model 2). Those identifying as gay/lesbian/bisexual/something else were 20% more likely to transition among tobacco product(s) than those identifying as heterosexual/straight after adjustment for all correlates (Table 1, Model 2, RRR = 1.2, 95% CI = 1.0–1.5). Tobacco users with higher nicotine dependence at W1 were more likely to transition among tobacco product(s) versus not transition than were those with nicotine dependence in the first quartile at W1 after adjustment for all correlates (Table 1, Model 2). Poly-tobacco users at W1 were about four times more likely to transition among tobacco product(s) versus not transition than were exclusive tobacco users at W1 after adjustment for all correlates (Table 1, Model 2, RRR = 3.9, 95% CI = 3.0–5.0).

Someday users versus non-users were more likely to transition among tobacco product(s) across models for each type of tobacco product except cigarettes, and those who were daily users versus non-users of smokeless tobacco were less likely to transition among tobacco product(s) (Table 1, Models 1–2).

#### 3.1.2. Correlates of Discontinuing Tobacco Use 

Young adults were more likely to discontinue tobacco use versus not transition than were older adults (Table 1, Model 1); however, the strength of this association was reduced/disappeared after adjustment for tobacco use correlates (Table 1, Model 2). Those identifying as gay/lesbian/bisexual/something else were 30% more likely to discontinue tobacco use than those identifying as heterosexual/straight after adjustment for all correlates (Table 1, Model 2, RRR = 1.3, 95% CI = 1.0–1.8). Tobacco users with higher nicotine dependence at W1 were less likely to discontinue tobacco use versus not transition than were those with nicotine dependence in the first quartile, and those who were daily users versus non-users of cigarettes, ENDS, or smokeless tobacco were also less likely to discontinue tobacco use, whereas those who were someday users versus non-users of cigars, pipe tobacco, or hookah were more likely to discontinue tobacco use regardless of adjustments (Table 1, Models 1–2). Associations for other demographic and tobacco use correlates among *any tobacco* users are shown in Table 1.

### 3.2. Exclusive Cigarette Smokers at W1 

Table 2 and Table 3 show rates and relative likelihood of transitions in tobacco product use among *exclusive cigarette* smokers at W1, as a function of correlates assessed at W1, using two separate approaches to transitions: Table 2 shows transitions only with respect to ENDS and Table 3 shows transitions with respect to all tobacco products.

When considering transitions only with respect to ENDS (Table 2), the majority of exclusive cigarette smokers, 78.2%, did not transition, 11.2% transitioned to cigarettes plus ENDS use, 1.5% switched to ENDS use, and 9.1% transitioned to no cigarettes and no ENDS use. When considering transitions with respect to all tobacco products (Table 3), the majority of exclusive cigarette smokers, 72.8%, did not transition, 6.3% transitioned to cigarettes plus combustible use, 10.3% transitioned to cigarettes plus non-combustible use, 1.9% switched to non-cigarette tobacco use, and 8.7% discontinued tobacco use.

#### 3.2.1. Correlates of Transitions to Cigarettes Plus ENDS Use (Table 2)

Among exclusive cigarette smokers, young adults were more than twice as likely to transition to cigarettes plus ENDS use versus not transition compared to those aged 55+ years (Table 2, Model 2, RRR = 0.4, 95% CI = 0.3–0.5), and about 1.4 times more likely to transition compared to those aged 40–54 years (Model 2, RRR = 0.7, 95% CI = 0.5–0.9), female smokers were more likely than male smokers (Model 2, RRR = 1.3 95% CI = 1.0–1.6), smokers who identified as gay/lesbian/bisexual/ something else were more likely than those who identified as heterosexual/straight (Model 2, RRR = 1.6, 95% CI = 1.1–2.3), and African American smokers were half as likely as non-Hispanic white smokers (Model 2, RRR = 0.5, 95% CI = 0.4–0.8), to transition to cigarettes plus ENDS use versus not transition regardless of adjustments (Table 2). Those in the third quartile of nicotine dependence at W1 were more likely than those in the first quartile to transition to cigarettes plus ENDS use regardless of adjustments (Table 2, Model 2, RRR = 1.8, 95% CI = 1.2–2.5), and daily cigarette smokers were more likely than someday cigarette smokers to transition to cigarettes plus ENDS use regardless of adjustments (Model 2, RRR = 1.5, 95% CI = 1.0–2.2).

#### 3.2.2. Correlates of Transitions to Cigarettes Plus Combustible Use (Table 3)

Among exclusive cigarette smokers, young adults were about 2–3 times more likely to transition to cigarettes plus combustible use versus not transition compared to their older adult counterparts regardless of adjustments (Table 3, Model 2 estimates ranging from RRR = 0.4, 95% CI = 0.3–0.6 for those aged 25–39 years and for those aged 40–54 years, to RRR = 0.3, 95% CI = 0.2–0.4 for those aged 55+ years), female smokers were about half as likely as male smokers (Model 2, RRR = 0.5, 95% CI = 0.4–0.6), and smokers who identified as gay/lesbian/bisexual/something else were about twice as likely as those who identified as heterosexual/straight (Model 2, RRR = 2.0, 95% CI = 1.2–3.3) to transition to cigarettes plus combustible use regardless of adjustments. Those in the third or fourth quartiles of nicotine dependence at W1 were more likely than those in the first quartile to transition to cigarettes plus combustible use regardless of adjustments (Table 3, Model 2, RRR = 1.7, 95% CI = 1.1–2.5 for third quartile; RRR = 1.7, 95% CI = 1.0–2.6 for fourth quartile). There was no difference in likelihood of transitioning to cigarettes plus combustible use between daily versus someday cigarette smokers at W1.

#### 3.2.3. Tobacco Product Switching (Table 2 and Table 3)

Among exclusive cigarette smokers, 1.5% switched to ENDS (alone or in combination with another non-cigarette tobacco product, Table 2) and 1.9% switched to any non-cigarette tobacco product use (which could include ENDS, Table 3). Most stratified estimates for switching transitions in Table 2 and Table 3 were based on a sample size of less than 50 or had a relative standard error of greater than 30% and are therefore unstable.

#### 3.2.4. Correlates of Transitions to No Cigarettes and No ENDS Use (Table 2) and to No Tobacco Use (Table 3)

Among exclusive cigarette smokers, African American smokers were less likely than non-Hispanic white smokers to transition to no cigarettes and no ENDS use (Table 2, Model 2, RRR = 0.6, 95% CI = 0.4–0.9), and to discontinue all tobacco use (Table 3, Model 2, RRR = 0.6, 95% CI = 0.4–0.9) when adjusting for all correlates. Those at or above twice the poverty level were 50% more likely than those below the poverty level to transition to no cigarettes and no ENDS use (Table 2, Model 2, RRR = 1.5, 95% CI = 1.1–2.1) and to discontinue all tobacco use (Table 3, Model 2, RRR = 1.5, 95% CI = 1.1–2.1) regardless of adjustments. Associations varied across adjustments for other demographic characteristics.

Exclusive cigarette smokers with higher nicotine dependence were far less likely than were those with nicotine dependence in the first quartile, and daily cigarette smokers were also far less likely than someday cigarette smokers, to transition to no cigarettes and no ENDS use (Table 2), and to discontinue all tobacco use (Table 3) regardless of adjustments.

### 3.3. Poly-Cigarette Smokers at W1

Table 4 shows rates and relative likelihood of transitions in tobacco product use among *poly-cigarette smokers* at W1, as a function of correlates assessed at W1. Overall, 54.5% of poly-cigarette smokers did not transition in use of tobacco products, 32.5% transitioned to exclusive cigarette use, 6.7% transitioned to non-cigarette tobacco use, and 6.3% discontinued tobacco use 

#### 3.3.1. Correlates of Transition to Exclusive Cigarette Use 

Among poly-cigarette smokers, young adults were about half as likely to transition to exclusive cigarette use versus not transition compared to those aged 40–54 years regardless of adjustments (Table 4, Model 2, RRR = 1.5, 95% CI = 1.1–1.9), female smokers were more likely than male smokers (Model 2, RRR = 1.4, 95% CI = 1.1–1.6), smokers with a bachelor’s degree or more were less likely than those with less than high school/GED education (Model 2, RRR = 0.6, 95% CI = 0.5–0.9), and those at or above twice the poverty level were more likely than those below the poverty level (Model 2, RRR = 1.5, 95% CI = 1.2–1.8), to transition to exclusive cigarette use regardless of adjustments. 

Those who were using three or more products at W1 were less likely than those using two products (Model 2, RRR = 0.6, 95% CI = 0.4–0.8), daily ENDS users were less likely than ENDS nonusers, daily cigar users were less likely than cigar non-users (Model 2, RRR = 0.3, 95% CI = 0.2–0.5 for ENDS use and for cigar use), and someday smokeless users were less likely than smokeless nonusers (Model 2, RRR = 0.6, 95% CI = 0.4–0.8) to transition to exclusive cigarette use versus not transition regardless of adjustments. 

#### 3.3.2. Correlates of Transition to Non-Cigarette Tobacco Use 

Among poly-cigarette smokers, those at or above the poverty level were more likely to transition to non-cigarette tobacco use compared to those below the poverty level regardless of adjustments (Model 2, RRR = 1.5, 95% CI = 1.0–2.3 for those at or near the poverty level; RRR = 2.0, 95% CI = 1.3–3.1 for those at or above twice the poverty level), and daily cigarette smokers were less likely than someday cigarette smokers to transition to non-cigarette tobacco use regardless of adjustments (Model 2, RRR = 0.4, 95% CI = 0.2–0.5). Associations varied across adjustments for other correlates (Table 4).

#### 3.3.3. Correlates of Discontinuing Tobacco Use

Among poly-cigarette smokers, female smokers were more likely than male smokers (Table 4, Model 2, RRR = 1.6, 95% CI = 1.1–2.3), Hispanic smokers were more likely than non-Hispanic white smokers (Model 2, RRR = 1.7, 95% CI = 1.0–2.7), and daily cigarette smokers were less likely than someday cigarette smokers (Model 2, RRR = 0.3, 95% CI = 0.2–0.5), to discontinue tobacco use versus not transition regardless of adjustments. Associations for other correlates varied across adjustments as shown in Table 4, including for number of products used at W1 and nicotine dependence at W1, both of which were negatively related to discontinuing tobacco use when adjusting only for demographic characteristics, but did not have robust and significant associations with discontinuing tobacco use after adjusting for all tobacco use variables.

## 4. Discussion

Data from W1 and W2 of the PATH Study indicate that transitions in tobacco product use among adult tobacco users, particularly young adults, are common, and that behaviors vary more and in different ways for different demographic groups and dependence levels, suggesting differing needs for tobacco control efforts among subgroups of the population. Among exclusive cigarette smokers, male smokers and those not identifying as heterosexual/straight were more likely to add other combustible tobacco product use to their cigarette use; female smokers, white smokers, and those not identifying as heterosexual/straight were more likely to add ENDS use to their cigarette use; and exclusive cigarette smokers below the poverty level were less likely to discontinue tobacco use than were their counterparts at or above twice the poverty level. Among poly-cigarette smokers, female smokers, those with higher education, and those with higher income were more likely to transition to exclusive cigarette use than their counterparts; and female smokers, and Hispanic smokers (compared to non-Hispanic white smokers), were more likely to discontinue tobacco use. These initial demographic findings can serve as a foundation for additional work that can help determine what drives demographic differences in tobacco use transitions. For example, differences in rates of exclusive cigarette smokers adding ENDS use found here may reflect demographic differences in quit intentions, as others have found that cigarette smokers report using ENDS to try to quit smoking [24,25].

Further, nicotine dependence remains a key barrier to discontinuing tobacco use. These findings show that those using tobacco on a daily basis and scoring higher on an index of nicotine dependence [20] were less likely to discontinue cigarette use and all tobacco use than their counterparts. Importantly, tobacco users with higher dependence scores were more likely to switch among tobacco products, and relatively highly dependent cigarette smokers were more likely to add product(s) to their cigarette use compared to their less dependent counterparts. Further research can elucidate these findings, but one possible explanation is that highly dependent cigarette smokers may be turning to other tobacco products as a means of trying to reduce or stop their cigarette use [24]. Limiting the amount of nicotine contained in cigarettes to reduce their addictiveness [26] has potential to mitigate the disease burden caused by combusted cigarettes, a high public health priority [27].

### Future Directions

These PATH Study findings provide a broad look at demographic and tobacco use characteristics associated with transitions in tobacco product use among current tobacco users over a 12-month period, and longer follow up can help us to better understand the effects of these transitions on public health. Additionally, as the variety of ENDS products available to consumers today expands, considering different types of ENDS products rather than considering all ENDS as one product could be useful for understanding transitions [3]. The PATH Study data can also be used to evaluate correlates of transitions among non-tobacco users (i.e., experimentation and initiation of tobacco use and relapse among former tobacco users), which will be important for determining the public health implications of the diverse tobacco product landscape. The work we present here can also be extended by evaluating changes in frequency of use within and among tobacco product types and further disaggregating transitions among poly-tobacco product users, all in an effort to help inform tobacco regulatory efforts aimed at protecting the health of the U.S. population.

## 5. Conclusions

Transitions in tobacco product use among adult tobacco users in the U.S. vary among different demographic groups, suggesting differing needs for tobacco control efforts among subgroups of the population. High nicotine dependence predicts transitioning among or the addition of tobacco products and is a barrier to discontinuing tobacco use, adding evidence to support policy to lower nicotine content of cigarettes and to evaluate new products for their potential to reduce cigarette use. 

## Figures and Tables

**Table 1:** Correlates of transitions in tobacco product use among ‘any tobacco’ users at Wave 1 (W1) of the Population Assessment of Tobacco and Health (PATH) Study ⱡ

	Transitions Assessed between W1 & Wave 2 (W2)												
	No transition (i.e., use the exact same tobacco product(s) at W2 as used at W1; referent group)			Transition among tobacco product(s) (i.e., any difference in use of tobacco product(s) at W1 and W2 except for the case of no tobacco use at W2)					Discontinue tobacco use (i.e., no use of any tobacco product at W2)				
							Model 1 ^1^	Model 2 ^2^				Model 1 ^1^	Model 2 ^2^
**Correlates assessed at W1**	N	%	SE	N	%	SE	RRR (95% CI)	RRR (95% CI)	N	%	SE	RRR (95% CI)	RRR (95% CI)
**Overall**	5947	47.9	0.55	5111	37.6	0.50			1804	14.5	0.37		
**Age group**													
18–24	942	27.0	0.84	1842	41.9	0.92	ref	ref	689	21.2	0.94	ref	ref
25–39	1745	43.9	0.89	1611	41.8	0.87	**0.5** **(0.5–0.6)**	**0.7** **(0.6–0.8)**	524	14.3	0.70	**0.4** **(0.3–0.5)**	0.9 (0.7–1.1)
40–54	1776	55.3	0.98	1079	33.6	0.85	**0.3** **(0.3–0.4)**	**0.5** **(0.5–0.6)**	316	11.1	0.64	**0.3** **(0.2–0.3)**	**0.7** **(0.6–0.9)**
55+	1483	63.0	1.14	578	23.8	1.00	**0.2** **(0.2–0.2)**	**0.4** **(0.3–0.4)**	275	13.3	0.86	**(0.2–0.4)**	0.8 (0.6–1.0)
Sex													
Male	3177	45.8	0.74	3071	39.8	0.67	ref	ref	968	14.3	0.50	ref	ref
Female	2768	51.0	0.87	2040	34.2	0.81	**0.7** **(0.7–0.8)**	0.9 (0.8–1.1)	834	14.7	0.52	0.9 (0.8–1.1)	**1.3** **(1.1–1.5)**
**Sexual orientation**													
Heterosexual/Straight	5500	48.7	0.57	4526	37.0	0.52	ref	ref	1614	14.3	0.38	ref	ref
Gay/lesbian/bisexual/something else	361	36.2	1.75	519	47.7	1.73	**1.5** **(1.3–1.8)**	**1.2** **(1.0–1.5)**	157	16.1	1.42	**1.3** **(1.0–1.6)**	**1.3** **(1.0–1.8)**
**Race/ethnicity**													
Non-Hispanic White	4052	50.0	0.68	3226	37.1	0.61	ref	ref	1009	12.9	0.43	ref	ref
Non-Hispanic Black or African American	839	48.7	1.51	692	37.0	1.37	1.0 (0.8–1.1)	0.9 (0.8–1.1)	266	14.3	1.22	**1.3** **(1.0–1.6)**	0.9 (0.7–1.1)
Non-Hispanic other	393	40.7	1.97	436	40.9	2.07	1.1 (0.9–1.4)	0.9 (0.7–1.2)	141	18.4	1.43	**1.5** **(1.2–1.8)**	1.2 (0.9–1.5)
Hispanic	663	38.6	1.17	757	39.4	1.45	1.1 (0.9–1.2)	1.0 (0.9–1.2)	388	22.0	1.32	**2.2** **(1.8–2.6)**	1.1 (0.9–1.4)
**Educational attainment**													
Less than high school or GED	1490	49.7	1.13	1326	40.7	1.13	ref	ref	284	9.6	0.61	ref	ref
High school graduate	1460	50.0	1.01	1282	37.6	1.03	0.9 (0.8–1.0)	0.9 (0.8–1.1)	395	12.5	0.59	**1.2** **(1.0–1.5)**	1.0 (0.8–1.2)
Some college (no degree) or associates degree	2126	45.6	0.86	1922	38.9	0.76	1.0 (0.9–1.1)	0.9 (0.8–1.0)	731	15.5	0.59	**1.6** **(1.3–2.0)**	1.1 (0.8–1.3)
Bachelor’s degree or more	871	46.8	1.34	581	30.6	1.11	0.8 (0.7–1.0)	**0.7** **(0.6–0.9)**	394	22.7	1.13	**2.5** **(2.0–3.2)**	0.9 (0.7–1.2)
**Poverty level**													
Below poverty level	1919	43.8	0.98	2068	43.6	0.83	ref	ref	582	12.6	0.62	ref	ref
At or near poverty level	1467	49.9	0.84	1159	37.3	0.86	0.9 (0.8–1.0)	1.0 (0.8–1.1)	377	12.9	0.67	1.0 (0.8–1.2)	1.0 (0.8–1.3)
At or above twice the poverty level	2097	49.2	0.89	1511	33.8	0.80	0.8 (0.7–1.0)	0.9 (0.8–1.1)	701	17.0	0.66	**1.2** **(1.0–1.4)**	1.0 (0.8–1.3)
Not reported	464	51.8	2.03	373	33.5	1.75	**0.7** **(0.6–0.9)**	0.9 (0.7–1.1)	144	14.8	1.37	1.0 (0.8–1.4)	1.0 (0.7–1.5)
**Total number of products currently used**													
1 product	4843	62.5	0.66	1708	20.2	0.53	ref	ref	1323	17.3	0.53	ref	ref
2 or more products	1104	24.5	0.78	3219	66.7	0.94	**7.4** **(6.6–8.3)**	**3.9** **(3.0–5.0)**	418	8.8	0.55	1.1 (1.0–1.3)	1.2 (0.8–1.8)
**Nicotine dependence ^3^**													
1st quartile	1200	37.1	0.89	1101	29.5	0.80	ref	ref	1086	33.3	0.82	ref	ref
2nd quartile	1468	52.0	1.08	1205	37.9	1.05	1.0 (0.9–1.2)	**1.2** **(1.0–1.4)**	308	10.1	0.59	**0.2** **(0.2–0.3)**	**0.5** **(0.4–0.7)**
3rd quartile	1771	53.4	1.07	1411	40.6	1.02	**1.2** **(1.0–1.3)**	**1.4** **(1.2–1.7)**	192	6.0	0.52	**0.2** **(0.1–0.2)**	**0.5** **(0.4–0.7)**
4th quartile	1411	51.4	1.10	1280	44.1	1.13	**1.4** **(1.3–1.7)**	**1.5** **(1.3–1.9)**	125	4.5	0.45	**0.1** **(0.1–0.2)**	**0.4** **(0.3–0.6)**
**Current use of cigarettes**													
No use	1469	39.7	0.90	1390	31.6	0.76	ref	ref	1142	28.8	0.83	ref	ref
Someday use	772	43.9	1.25	711	36.3	1.21	1.1 (0.9–1.2)	0.8 (0.6–1.1)	339	19.8	1.13	**0.6** **(0.5–0.7)**	**0.7** **(0.5–1.0)**
Daily use	3706	53.7	0.74	3002	41.4	0.72	**1.2** **(1.0–1.3)**	0.8 (0.6–1.0)	322	4.9	0.30	**0.1** **(0.1–0.2)**	**0.2** **(0.1–0.3)**
**Current use of ENDS**													
No use	5156	53.1	0.62	3340	31.5	0.55	ref	ref	1486	15.4	0.43	ref	ref
Someday use	535	24.1	1.00	1477	63.5	1.08	**4.4** **(3.8–5.0)**	**1.6** **(1.2–2.1)**	277	12.4	0.75	**1.7** **(1.4–2.0)**	1.4 (1.0–1.9)
Daily use	256	46.1	2.39	287	47.6	2.27	**1.9** **(1.5–2.3)**	1.1 (0.8–1.6)	36	6.3	1.09	**0.5** **(0.4–0.8)**	**0.3** **(0.2–0.5)**
**Current use of traditional cigars/cigarillos/filtered cigars**													
No use	5033	57.5	0.62	2757	29.3	0.56	ref	ref	1166	13.2	0.42	ref	ref
Someday use	772	25.8	0.94	1980	56.4	1.05	**4.2** **(3.8–4.8)**	**2.0** **(1.6–2.5)**	546	17.9	0.74	**2.8** **(2.4–3.2)**	**1.4** **(1.1–1.9)**
Daily use	142	33.6	2.69	267	55.6	2.66	**3.7** **(2.8–4.9)**	**1.6** **(1.2–2.2)**	53	10.9	1.67	**1.7** **(1.1–2.5)**	1.1(0.7–1.8)
**Current use of pipe**													
No use	5864	49.3	0.57	4690	36.2	0.49	ref	ref	1740	14.5	0.40	ref	ref
Someday use	65	14.6	1.86	385	70.6	2.50	**6.2** **(4.4–8.7)**	**2.1** **(1.4–3.3)**	60	14.8	2.00	**3.3** **(2.1–5.2)**	**1.7** **(1.1–2.8)**
Daily use	18	40.7	7.14	28	55.3	7.98	**2.4** **(1.3–4.5)**	1.4 (0.6–3.3)	2	4.0	2.76	0.4 (0.1–2.2)	0.3 (0.0–1.7)
**Current use of hookah**													
No use	5577	54.1	0.61	3649	33.5	0.52	ref	ref	1208	12.4	0.35	ref	ref
Someday use	361	15.6	0.86	1430	59.1	1.31	**4.4** **(3.8–5.1)**	**2.7** **(2.1–3.5)**	585	25.4	1.07	**4.3** **(3.6–5.1)**	**2.3** **(1.6–3.3)**
Daily use	9	21.9	6.80	29	62.2	7.79	**3.5** **(1.3–9.3)**	1.7 (0.6–5.1)	7	15.9	6.30	2.2 (0.8–8.4)	1.1 (0.2–5.6)
**Current use of smokeless/snus pouches/dissolvable**													
No use	5276	48.6	0.58	4267	36.3	0.54	ref	ref	1636	15.1	0.39	ref	ref
Someday use	180	25.5	1.76	516	62.4	1.93	**2.8** **(2.3–3.4)**	**1.6** **(1.3–2.0)**	92	12.1	1.27	**1.4** **(1.1–1.9)**	1.1 (0.8–1.6)
Daily use	491	62.9	1.76	267	28.9	1.59	**0.6** **(0.5–0.7)**	**0.6** **(0.5–0.7)**	59	8.2	1.18	**0.5** **(0.3–0.6)**	**0.4** **(0.2–0.5)**

ⱡ Table 1 notes. Sample includes adults aged 18+ years who were ‘any tobacco’ users at W1 and also participated in W2 (N = 12,862); analyses weighted using W2 longitudinal weights**.** Results from multinomial logistic regression analyses indicate the relative probability of being in each W2 transition group vs the W2 ‘no transition’ group as a function of correlates assessed at W1. Current use at each wave defined for cigarettes as having smoked at least 100 cigarettes in lifetime and now smoking everyday or somedays; for hookah as now using everyday, somedays, or monthly; for all other products as now using everyday or somedays. Bold font indicates significant association at α = 0.05; grey shaded cells indicate unstable estimate due to denominator <50 or relative standard of the estimate >30%**.** ^1^ Analyses adjusted for age, sex, sexual orientation, race/ethnicity, education, and poverty level. ^2^ Analyses adjusted for age, sex, sexual orientation, race/ethnicity, education, poverty level, total number of products used, nicotine dependence, and type and frequency of product used. ^3^ Mean cumulative nicotine dependence score; quartiles determined among W1 ‘any tobacco’ users.

**Table 2:** Correlates of transitions in tobacco product use among ‘exclusive cigarette’ smokers at Wave 1 (W1); transitions defined only with respect to cigarettes and Electronic Nicotine Delivery Systems (ENDS) use ⱡ.

	Transitions Assessed between W1 & Wave 2 (W2)																	
	No transition ^a^ (i.e., cigarette use at W2, no ENDS use at W2; referent group)			Transition to cigarettes plus ENDS use (i.e., cigarette use at W2 and ENDS use at W2)					Switch to ENDS use (i.e., no cigarette use at W2, ENDS use at W2)					Transition to no cigarette and no ENDS use (i.e., no cigarette use at W2, no ENDS use at W2)				
							Model 1 ^1^	Model 2 ^2^				Model 1 ^1^	Model 2 ^2^				Model 1 ^1^	Model 2 ^2^
**Correlates assessed at W1**	N	%	SE	N	%	SE	RRR (95% CI)	RRR (95% CI)	N	%	SE	RRR (95% CI)	RRR (95% CI)	N	%	SE	RRR (95% CI)	RRR (95% CI)
**Overall**	3846	78.2	0.66	558	11.2	0.52			75	1.5	0.20			414	9.1	0.46		
**Age group**																		
18–24	489	70.9	1.72	104	15.3	1.49	ref	ref	12	1.9	0.61	ref	ref	74	12.0	1.20	ref	ref
25–39	1166	75.1	1.28	189	13.0	1.05	0.8 (0.6–1.1)	0.8 (0.6–1.0)	38	2.5	0.46	1.2 (0.5–2.7)	1.1 (0.5–2.5)	134	9.5	0.97	**0.7** **(0.5–0.9)**	0.8 (0.6–1.1)
40–54	1186	79.9	1.23	189	12.0	0.98	**0.7** **(0.6–1.0)**	**0.7** **(0.5–0.9)**	14	1.0	0.28	0.4 (0.2–1.2)	0.4 (0.1–1.1)	99	7.1	0.86	**0.5** **(0.3–0.7)**	0.8 (0.5–1.1)
55+	1005	82.3	1.28	76	6.6	0.74	**0.4** **(0.3–0.6)**	**0.4** **(0.3–0.5)**	11	0.9	0.31	0.4 (0.2–1.1)	0.4 (0.1–1.1)	107	10.1	1.07	0.7 (0.5–1.0)	1.1 (0.8–1.7)
Sex																		
Male	1708	79.3	0.90	207	9.7	0.70	ref	ref	40	1.8	0.30	ref	ref	182	9.2	0.72	ref	ref
Female	2138	77.2	0.97	351	12.6	0.73	**1.3** **(1.1–1.6)**	**1.3** **(1.0–1.6)**	35	1.2	0.25	0.6 (0.4–1.1)	0.6 (0.4–1.0)	231	9.0	0.69	1.1 (0.8–1.4)	1.1 (0.8–1.5)
**Sexual orientation**																		
Heterosexual/Straight	3544	78.5	0.69	495	10.8	0.52	ref	ref	72	1.6	0.22	ref	ref	377	9.1	0.48	ref	ref
Gay/lesbian/bisexual/something else	239	71.9	3.05	58	18.0	2.57	**1.6** **(1.1–2.4)**	**1.6** **(1.1–2.3)**	2	0.3	0.25	0.2 (0.0–1.3)	0.2 (0.0–1.3)	30	9.9	2.13	1.1 (0.7–1.9)	1.4 (0.8–2.3)
**Race/ethnicity**																		
Non-Hispanic White	2519	77.2	0.77	421	12.3	0.63	ref	ref	60	1.8	0.29	ref	ref	258	8.6	0.53	ref	ref
Non-Hispanic Black or African American	639	86.1	1.43	46	6.5	1.00	**0.5** **(0.3–0.7)**	**0.5** **(0.4–0.8)**	5	0.8	0.37	0.5 (0.1–1.5)	0.5 (0.1–1.5)	49	6.6	1.18	0.8 (0.6–1.2)	**0.6** **(0.4–0.9)**
Non-Hispanic other	233	69.8	3.37	37	13.3	2.50	1.1 (0.7–1.9)	1.2 (0.7–2.0)	4	0.6	0.32	0.3 (0.1–1.2)	0.3 (0.1–1.3)	35	16.3	2.63	**1.9** **(1.3–2.9)**	1.5 (1.0–2.3)
Hispanic	455	78.8	1.85	54	8.3	1.24	**0.7** **(0.5–0.9)**	0.8 (0.6–1.1)	6	1.0	0.35	0.6 (0.2–1.4)	0.6 (0.2–1.5)	72	11.9	1.54	**1.6** **(1.1–2.2)**	0.9 (0.6–1.2)
**Educational attainment**																		
Less than high school or GED	1160	81.4	1.06	157	10.9	0.91	ref	ref	13	0.8	0.26	ref	ref	86	6.9	0.82	ref	ref
High school graduate	981	79.6	1.13	142	10.8	0.96	0.9 (0.7–1.2)	1.0 (0.8–1.3)	14	1.2	0.36	1.4 (0.6–3.4)	1.4 (0.6–3.5)	101	8.3	0.71	1.2 (0.9–1.6)	1.0 (0.7–1.3)
Some college (no degree) or associates degree	1313	76.5	1.22	202	11.6	0.88	0.9 (0.7–1.3)	1.0 (0.7–1.3)	40	2.4	0.42	**2.5** **(1.1–5.7)**	**2.5** **(1.1–5.7)**	152	9.4	0.85	1.3 (0.9–1.8)	1.0 (0.7–1.4)
Bachelor’s degree or more	392	71.8	2.19	57	11.3	1.34	1.0 (0.7–1.4)	1.1 (0.8–1.6)	8	1.3	0.41	1.5 (0.5–4.0)	1.6 (0.5–4.6)	75	15.6	1.79	**2.0** **(1.3–2.9)**	1.0 (0.7–1.6)
**Poverty level**																		
Below poverty level	1421	80.0	1.04	205	11.3	0.80	ref	ref	23	1.2	0.19	ref	ref	125	7.6	0.72	ref	ref
At or near poverty level	1025	79.9	1.18	147	11.2	0.92	1.0 (0.8–1.4)	1.0 (0.8–1.4)	23	1.8	0.41	1.3 (0.7–2.2)	1.2 (0.7–2.1)	93	7.1	0.81	0.9 (0.7–1.3)	1.0 (0.7–1.4)
At or above twice the poverty level	1091	74.7	1.37	165	11.4	1.01	1.1 (0.8–1.5)	1.1 (0.8–1.5)	26	1.9	0.41	1.2 (0.7–2.2)	1.3 (0.7–2.3)	160	12.1	0.97	**1.5** **(1.1–2.1)**	**1.5** **(1.1–2.1)**
Not reported	309	79.6	2.46	41	10.1	1.76	1.0 (0.7–1.6)	1.1 (0.7–1.7)	3	0.6	0.36	0.6 (0.1–2.7)	0.6 (0.1–2.9)	36	9.7	1.77	1.3 (0.8–2.1)	1.4 (0.8–2.3)
**Nicotine dependence ^3^**																		
1st quartile	915	72.5	1.37	95	6.5	0.79	ref	ref	19	1.5	0.33	ref	ref	229	19.6	1.19	ref	ref
2nd quartile	932	80.4	1.31	128	11.2	0.98	**1.6** **(1.1–2.2)**	1.4 (0.9–2.0)	15	1.3	0.35	0.9 (0.4–2.0)	0.8 (0.3–1.8)	79	7.2	0.97	**0.3** **(0.2–0.5)**	**0.5** **(0.4–0.8)**
3rd quartile	993	78.7	1.21	184	14.8	1.02	**2.1** **(1.5–2.9)**	**1.8** **(1.2–2.5)**	24	2.0	0.48	1.4 (0.7–3.0)	1.2 (0.5–2.7)	51	4.5	0.64	**0.2** **(0.2–0.3)**	**0.4** **(0.3–0.7)**
4th quartile	951	81.6	1.24	149	12.8	1.08	**1.8** **(1.2–2.5)**	1.5 (1.0–2.2)	16	1.4	0.39	1.0 (0.4–2.5)	0.9 (0.3–2.3)	48	4.2	0.72	**0.2** **(0.1–0.3)**	**0.4** **(0.3–0.6)**
**Current use of cigarettes**																		
Someday use	627	67.9	1.77	60	6.0	0.85	ref	ref	14	1.3	0.30	ref	ref	209	24.8	1.62	ref	ref
Daily use	3219	80.6	0.68	498	12.4	0.59	**1.9** **(1.3–2.7)**	**1.5** **(1.0–2.2)**	61	1.6	0.25	1.4 (0.7–2.6)	1.4 (0.7–2.7)	205	5.5	0.43	**0.2** **(0.1–0.2)**	**0.3** **(0.2–0.4)**

ⱡ Table 2 notes. Sample includes adults aged 18+ years who were ‘exclusive cigarette’ smokers at W1 and also participated in W2 (N = 4893); analyses weighted using W2 longitudinal weights. Results from multinomial logistic regression analyses indicate the relative probability of being in each W2 transition group vs the W2 ‘no transition’ group as a function of correlates assessed at W1. Current use at each wave defined for cigarettes as having smoked at least 100 cigarettes in lifetime and now smoking everyday or somedays; for hookah as now using everyday, somedays, or monthly; for all other products as now using everyday or somedays. ‘Exclusive cigarette’ use defined as current use of cigarettes and no current use of any other tobacco product. Bold font indicates significant association at α = 0.05; grey shaded cells indicate unstable estimate due to denominator <50 or relative standard of the estimate >30%. ^1^ Analyses adjusted for age, sex, sexual orientation, race/ethnicity, education, and poverty level. ^2^ Analyses adjusted for age, sex, sexual orientation, race/ethnicity, education, poverty level, tobacco dependence, and frequency of cigarette use. ^3^ Mean cumulative tobacco dependence score; quartiles determined among W1 ‘exclusive cigarette’ smokers. ^a^ No transition is defined only with respect to cigarettes and ENDS use; therefore, no transition may include adding combustible or smokeless tobacco products(s) at W2.

**Table 3:** Correlates of transitions in tobacco product use among ‘exclusive cigarette’ smokers at Wave 1 (W1); transitions defined with respect to all tobacco products ⱡ.

	Transitions Assessed between W1 & Wave 2 (W2)																						
	No transition ^a^ (i.e., exclusive cigarette use at W2; referent group)			Transition to cigarettes plus combustible use (i.e., cigarette use and use of at least one other combustible tobacco product at W2; may also use any non-combustible tobacco product(s) at W2)					Transition to cigarettes plus non-combustible use only (i.e., cigarette use and use of at least one non-combustible tobacco product at W2; does not use any combustible tobacco product at W2)					Switch to non-cigarette tobacco use (i.e., no cigarette use at W2, but use of any other tobacco product at W2)					Discontinue tobacco use (i.e., no use of any tobacco product at W2)				
							Model 1 ^1^	Model 2 ^2^				Model 1 ^1^	Model 2 ^2^				Model 1 ^1^	Model 2 ^2^				Model 1 ^1^	Model 2 ^2^
**Correlates assessed at W1**	N	%	SE	N	%	SE	RRR (95% CI)	RRR (95% CI)	N	%	SE	RRR (95% CI)	RRR (95% CI)	N	%	SE	RRR (95% CI)	RRR (95% CI)	N	%	SE	RRR (95% CI)	RRR (95% CI)
**Overall**	3524	72.8	0.70	341	6.3	0.39			496	10.3	0.55			92	1.9	0.20			393	8.7	0.45		
**Age group**																							
18–24	404	60.8	2.01	91	13.2	1.31	ref	ref	77	11.9	1.37	ref	ref	17	2.7	0.73	ref	ref	67	11.4	1.31	ref	ref
25–39	1064	69.5	1.31	107	6.7	0.68	**0.5** **(0.3–0.6)**	**0.4** **(0.3–0.6)**	170	12.0	1.05	0.9 (0.7–1.3)	0.9 (0.6–1.2)	43	2.9	0.53	0.9 (0.4–1.9)	0.9 (0.4–1.8)	127	8.9	0.93	**0.6** **(0.4–0.8)**	0.8 (0.5–1.1)
40–54	1109	74.7	1.25	88	5.9	0.71	**0.4** **(0.3–0.6)**	**0.4** **(0.3–0.6)**	174	11.3	1.03	0.8 (0.6–1.1)	**0.7** **(0.5–1.0)**	16	1.2	0.27	**0.3** **(0.1–0.8)**	**0.3** **(0.1–0.7)**	97	7.0	0.84	**0.5** **(0.3–0.7)**	0.7 (0.5–1.1)
55+	947	78.1	1.27	55	4.2	0.68	**0.3** **(0.2–0.4)**	**0.3** **(0.2–0.4)**	75	6.7	0.72	**0.5** **(0.3–0.7)**	**0.4** **(0.3–0.6)**	16	1.4	0.34	**0.4** **(0.2–0.8)**	**0.4** **(0.2–0.8)**	102	9.6	1.05	**0.6** **(0.4–1.0)**	1.1 (0.7–1.6)
Sex																							
Male	1513	71.6	0.99	192	8.2	0.64	ref	ref	189	9.2	0.72	ref	ref	49	2.3	0.32	ref	ref	172	8.8	0.69	ref	ref
Female	2011	73.9	0.99	149	4.6	0.37	**0.5** **(0.4–0.7)**	**0.5** **(0.4–0.6)**	307	11.4	0.74	1.2 (0.9–1.4)	1.1 (0.9–1.4)	43	1.6	0.30	0.7 (0.4–1.1)	0.6 (0.4–1.1)	220	8.6	0.67	1.0 (0.8–1.3)	1.1 (0.8–1.4)
**Sexual orientation**																							
Heterosexual/Straight	3262	73.2	0.73	299	6.0	0.39	ref	ref	443	10.1	0.58	ref	ref	88	2.0	0.22	ref	ref	358	8.7	0.47	ref	ref
Gay/lesbian/bisexual/something else	205	63.2	3.17	36	11.1	2.07	**2.0** **(1.2–3.2)**	**2.0** **(1.2–3.3)**	49	15.8	2.52	**1.7** **(1.1–2.5)**	**1.6** **(1.1–2.5)**	3	0.6	0.34	0.3 (0.1–1.2)	0.3 (0.1–1.3)	28	9.4	2.09	1.2 (0.7–2.0)	1.5 (0.9–2.5)
**Race/ethnicity**																							
Non-Hispanic White	2332	72.2	0.78	198	5.7	0.45	ref	ref	387	11.8	0.67	ref	ref	68	2.1	0.28	ref	ref	246	8.3	0.53	ref	ref
Non-Hispanic Black or African American	563	78.2	1.81	77	9.3	1.06	1.3 (1.0–1.7)	1.4 (1.0–1.9)	33	4.9	0.84	**0.4** **(0.3–0.6)**	**0.4** **(0.3–0.7)**	11	1.6	0.53	0.8 (0.4–1.9)	0.9 (0.4–2.0)	44	6.0	1.10	0.8 (0.5–1.2)	**0.6** **(0.4–0.9)**
Non-Hispanic other	209	64.8	3.53	27	7.2	1.60	1.2 (0.7–2.1)	1.2 (0.7–2.1)	30	10.9	2.36	1.0 (0.6–1.7)	1.1 (0.6–1.8)	6	2.0	1.29	0.9 (0.2–3.9)	0.9 (0.2–3.8)	33	15.1	2.66	**1.9** **(1.2–2.9)**	1.5 (0.9–2.3)
Hispanic	420	73.7	2.11	39	6.0	1.15	0.8 (0.5–1.4)	0.9 (0.5–1.5)	46	7.5	1.18	0.6 (0.4–2.9)	0.7 (0.5–1.1)	7	1.1	0.38	0.6 (0.2–1.3)	0.6 (0.3–1.3)	70	11.7	1.55	**1.6** **(1.1–2.2)**	0.9 (0.6–1.3)
**Educational attainment**																							
Less than high school or GED	1042	74.1	1.12	116	7.8	0.74	ref	ref	145	10.3	0.82	ref	ref	16	1.1	0.28	ref	ref	83	6.7	0.79	ref	ref
High school graduate	907	74.6	1.34	80	6.0	0.81	0.8(0.6–1.2)	0.9 (0.6–1.3)	123	10.1	1.00	0.9 (0.7–1.1)	0.9 (0.7–1.2)	19	1.7	0.40	1.4 (0.7–2.9)	1.4 (0.7–3.0)	93	7.7	0.74	1.1 (0.8–1.5)	0.9 (0.6–1.2)
Some college (no degree) or associates degree	1208	71.9	1.29	112	5.6	0.64	0.8 (0.6–1.1)	0.8 (0.6–1.2)	178	10.6	0.94	0.9 (0.7–1.2)	0.9 (0.7–1.2)	45	2.7	0.43	**2.1** **(1.1–4.3)**	**2.1** **(1.0–4.2)**	146	9.2	0.87	1.2 (0.9–1.8)	0.9 (0.6–1.3)
Bachelor’s degree or more	367	67.2	2.20	33	6.0	1.18	1.1 (0.7–1.7)	1.1 (0.7–1.9)	50	9.9	1.36	0.8 (0.6–1.3)	1.0 (0.6–1.5)	12	2.3	0.76	2.0 (0.9–4.5)	2.0 (0.9–4.7)	71	14.5	1.72	**1.8** **(1.2–2.8)**	0.9 (0.6–1.5)
**Poverty level**																							
Below poverty level	1276	72.9	1.29	164	8.6	0.77	ref	ref	169	9.6	0.76	ref	ref	28	1.5	0.25	ref	ref	120	7.4	0.72	ref	ref
At or near poverty level	942	74.7	1.26	76	5.7	0.70	0.7 (0.5–1.0)	0.7 (0.5–1.1)	137	10.8	0.97	1.2 (0.9–1.5)	1.2 (0.9–1.5)	28	2.2	0.42	1.3 (0.8–2.3)	1.3 (0.8–2.2)	85	6.6	0.76	0.9 (0.6–1.2)	0.9 (0.6–1.3)
At or above twice the poverty level	1022	70.4	1.35	76	4.9	0.64	0.7 (0.4–1.0)	0.7 (0.4–1.0)	152	10.8	1.00	1.2 (0.9–1.6)	1.2 (0.9–1.6)	30	2.2	0.46	1.2 (0.6–2.2)	1.2 (0.7–2.3)	156	11.8	0.98	**1.5** **(1.1–2.1)**	**1.5** **(1.1–2.1)**
Not reported	284	75.2	2.78	25	5.2	1.08	0.7 (0.4–1.1)	0.7 (0.4–1.1)	38	9.7	1.71	1.1 (0.7–1.7)	1.2 (0.8–1.9)	6	1.4	0.56	1.1 (0.4–2.9)	1.2 (0.4–3.1)	32	8.5	1.71	1.1 (0.6–1.9)	1.2 (0.7–2.1)
**Nicotine dependence ^3^**																							
1st quartile	842	68.1	1.46	72	4.8	0.65	ref	ref	85	6.0	0.76	ref	ref	25	1.8	0.38	ref	ref	221	19.3	1.21	ref	ref
2nd quartile	860	75.1	1.46	78	6.4	0.76	1.3 (0.8–1.9)	1.4 (0.9–2.1)	112	10.1	0.96	**1.5** **(1.0–2.1)**	1.3 (0.9–1.8)	20	1.9	0.46	1.1 (0.5–2.3)	1.1 (0.4–2.8)	73	6.6	0.87	**0.3** **(0.2–0.4)**	**0.5** **(0.3–0.7)**
3rd quartile	904	72.6	1.30	94	7.1	0.70	**1.5** **(1.1–2.1)**	**1.7** **(1.1–2.5)**	168	14.0	1.04	**2.1** **(1.5–3.0)**	**1.7** **(1.2–2.5)**	26	2.2	0.50	1.4 (0.7–2.7)	1.5 (0.7–3.2)	47	4.1	0.67	**0.2** **(0.1–0.3)**	**0.4** **(0.3–0.6)**
4th quartile	871	75.5	1.37	88	6.9	0.73	**1.5** **(1.0–2.3)**	**1.7** **(1.0–2.6)**	130	11.9	1.22	**1.7** **(1.2–2.5)**	1.4 (0.9–2.1)	20	1.8	0.43	1.2 (0.6–2.5)	1.3 (0.5–3.3)	45	3.9	0.64	**0.2** **(0.1–0.3)**	**0.4** **(0.2–0.6)**
**Current use of cigarettes**																							
Someday use	569	63.2	1.75	58	5.7	0.83	ref	ref	49	5.0	0.76	ref	ref	19	2.1	0.45	ref	ref	202	24.1	1.69	ref	ref
Daily use	2955	75.0	0.73	283	6.5	0.44	1.1 (0.7–1.5)	0.8 (0.5–1.3)	447	11.5	0.64	**2.1** **(1.4–3.0)**	**1.7** **(1.1–2.5)**	73	1.9	0.24	1.0 (0.6–1.8)	0.9 (0.4–1.8)	191	5.1	0.42	**0.2** **(0.1–0.2)**	**0.3** **(0.2–0.4)**

ⱡ Table 3 notes. Sample includes adults aged 18+ years who were ‘exclusive cigarette’ smokers at W1 and also participated in W2 (N = 4893); analyses weighted using W2 longitudinal weights. Results from multinomial logistic regression analyses indicate the relative probability of being in each W2 transition group vs the W2 ‘no transition’ group as a function of correlates assessed at W1. Current use at each wave defined for cigarettes as having smoked at least 100 cigarettes in lifetime and now smoking everyday or somedays; for hookah as now using everyday, somedays, or monthly; for all other products as now using everyday or somedays. ‘Exclusive cigarette’ use defined as current use of cigarettes and no current use of any other tobacco product. Bold font indicates significant association at α = 0.05; grey shaded cells indicate unstable estimate due to denominator <50 or relative standard of the estimate >30%. ^1^ Analyses adjusted for age, sex, sexual orientation, race/ethnicity, education, and poverty level. ^2^ Analyses adjusted for age, sex, sexual orientation, race/ethnicity, education, poverty level, tobacco dependence, and frequency of cigarette use. ^3^ Mean cumulative tobacco dependence score; quartiles determined among W1 ‘exclusive cigarette’ smokers. ^a^ No transition is defined only with respect to cigarettes and ENDS use; therefore, no transition may include adding combustible or smokeless tobacco products(s) at W2.

**Table 4:** Correlates of transitions in tobacco product use among ‘poly cigarette’ smokers at Wave 1 (W1) ⱡ.

	Transitions assessed between Wave 1 & Wave 2 (W2)																	
	No transition ^a^ (i.e., poly cigarette use at W2; referent group)			Transition to exclusive cigarette (i.e., cigarette use at W2, no use of any other tobacco product at W2)					Transition to non-cigarette tobacco use (i.e., no cigarette use at W2, but use of any other tobacco product(s) at W2; 56.4% of people in this transition group were ENDS users at W2)					Discontinue tobacco use (i.e., no use of any tobacco product at W2)				
							Model 1 ^1^	Model 2 ^2^				Model 1 ^1^	Model 2 ^2^				Model 1 ^1^	Model 2 ^2^
**Correlates assessed at W1**	N	%	SE	N	%	SE	RRR (95% CI)	RRR (95% CI)	N	%	SE	RRR (95% CI)	RRR (95% CI)	N	%	SE	RRR (95% CI)	RRR (95% CI)
**Overall**	2195	54.5	0.92	1248	32.5	0.88			261	6.7	0.49			242	6.3	0.46		
**Age group**																		
18–24	719	60.2	1.45	292	23.5	1.35	ref	ref	93	8.0	0.80	ref	ref	88	8.4	1.02	ref	ref
25–39	740	55.7	1.52	427	31.6	1.40	**1.4** **(1.1–1.7)**	1.2 (1.0–1.6)	85	6.6	0.87	0.8 (0.5–1.1)	1.0 (0.6–1.5)	84	6.0	0.67	0.8 (0.5–1.1)	1.0 (0.7–1.4)
40–54	479	50.4	1.78	346	39.0	1.66	**1.8** **(1.4–2.2)**	**1.5** **(1.1–1.9)**	49	5.7	0.91	0.7 (0.5–1.1)	0.9 (0.5–1.4)	41	4.9	0.75	0.7 (0.4–1.1)	0.8 (0.5–1.4)
55+	256	50.2	2.50	182	36.4	2.31	**1.7** **(1.3–2.3)**	1.3 (0.9–1.9)	34	6.9	1.24	0.9 (0.6–1.4)	1.1 (0.7–1.8)	29	6.6	1.38	0.9 (0.5–1.7)	1.2 (0.6–2.4)
**Sex**																		
Male	1405	58.1	1.19	629	28.5	1.02	ref	ref	191	7.9	0.62	ref	ref	123	5.5	0.58	ref	ref
Female	790	48.2	1.47	619	39.4	1.54	**1.8** **(1.5–2.1)**	**1.4** **(1.1–1.6)**	70	4.6	0.67	0.8 (0.5–1.1)	0.8 (0.6–1.2)	119	7.7	0.78	**1.7** **(1.2–2.3)**	**1.6** **(1.1–2.3)**
**Sexual orientation**																		
Heterosexual/Straight	1938	54.6	1.00	1106	32.5	0.89	ref	ref	235	6.9	0.52	ref	ref	202	6.0	0.48	ref	ref
Gay/lesbian/bisexual/something else	244	57.8	2.58	117	29.7	2.64	0.8 (0.6–1.0)	0.9 (0.6–1.2)	23	4.8	1.26	0.7 (0.4–1.3)	0.7 (0.4–1.4)	33	7.7	1.36	1.0 (0.6–1.6)	1.1 (0.6–1.7)
**Race/ethnicity**																		
Non-Hispanic White	1485	55.3	1.12	813	32.2	1.07	ref	ref	180	7.1	0.67	ref	ref	137	5.4	0.51	ref	ref
Non-Hispanic Black or African American	253	53.5	2.67	168	36.4	2.45	1.2 (0.9–1.6)	1.1 (0.8–1.4)	24	5.0	1.20	0.9 (0.5–1.6)	0.7 (0.4–1.4)	27	5.1	1.12	1.1 (0.7–1.9)	0.8 (0.4–1.4)
Non-Hispanic other	202	57.0	3.66	99	28.4	3.46	1.0 (0.7–1.4)	0.8 (0.6–1.3)	17	4.4	1.12	0.6 (0.3–1.1)	0.5 (0.3–1.0)	25	10.2	2.30	**2.0** **(1.1–3.4)**	1.7 (0.9–3.0)
Hispanic	255	48.9	2.50	168	32.9	2.32	1.2 (1.0–1.6)	1.1 (0.8–1.4)	40	7.4	1.12	1.4 (0.9–2.1)	1.1 (0.7–1.9)	53	10.8	1.55	**2.6** **(1.7–4.0)**	**1.7** **(1.0–2.7)**
**Educational attainment**																		
Less than high school or GED	591	54.9	1.46	361	35.7	1.50	ref	ref	48	4.6	0.84	ref	ref	43	4.8	0.82	ref	ref
High school graduate	549	54.8	1.96	299	33.2	1.95	0.9 (0.7–1.2)	0.9 (0.7–1.1)	67	7.2	1.06	1.5 (0.9–2.6)	1.4 (0.8–2.5)	48	4.7	0.75	1.0 (0.6–1.6)	0.8 (0.5–1.4)
Some college (no degree) or associates degree	835	55.0	1.39	466	30.7	1.32	**0.8** **(0.6–0.9)**	**0.7** (0.6–1.9)	111	7.1	0.71	1.4 (0.9–2.4)	1.2 (0.7–2.0)	111	7.2	0.81	1.3 (0.8–2.2)	1.1 (0.7–1.9)
Bachelor’s degree or more	220	51.3	2.89	122	29.5	2.54	**0.7** **(0.5–0.9)**	**0.6** **(0.5–0.9)**	35	8.8	1.35	1.7 (0.9–3.2)	1.1 (0.5–2.2)	40	10.4	1.59	**1.8** **(1.1–3.1)**	1.1 (0.6–2.1)
**Poverty level**																		
Below poverty level	935	57.8	1.21	508	32.5	1.29	ref	ref	78	4.6	0.52	ref	ref	81	5.1	0.59	ref	ref
At or near poverty level	529	55.5	1.63	291	30.8	1.49	1.1 (0.9–1.3)	1.0 (0.8–1.3)	75	7.3	0.95	**1.6** **(1.1–2.3)**	**1.5** **(1.0–2.3)**	57	6.4	0.93	1.4 (0.9–2.1)	1.3 (0.8–2.1)
At or above twice the poverty level	575	49.1	1.64	374	34.9	1.78	**1.4** **(1.2–1.8)**	**1.5** **(1.2–1.8)**	93	8.9	1.14	**2.1** **(1.4–3.2)**	**2.0** **(1.3–3.1)**	82	7.1	0.91	**1.7** **(1.1–2.5)**	1.5 (0.9–2.3)
Not reported	156	58.5	3.93	75	27.7	3.53	0.8 (0.5–1.3)	0.8 (0.5–1.2)	15	5.5	1.49	1.0 (0.5–2.2)	1.3 (0.6–2.7)	22	8.3	1.97	1.4 (0.8–2.7)	1.5 (0.7–3.0)
**Total number of products currently used**																		
2 products	1191	48.1	1.15	934	38.9	1.17	ref	ref	146	6.2	0.64	ref	ref	160	6.8	0.64	ref	ref
3 or more products	927	67.6	1.40	259	19.3	1.25	**0.4** **(0.3–0.5)**	**0.6** **(0.4–0.8)**	105	7.8	0.84	0.9 (0.6–1.3)	1.0 (0.5–2.1)	75	5.3	0.61	**0.5** **(0.4–0.8)**	0.5 (0.2–1.0)
**Nicotine dependence ^3^**																		
1st quartile	539	50.5	1.82	288	28.2	1.63	ref	ref	99	10.3	1.17	ref	ref	112	11.1	1.16	ref	ref
2nd quartile	496	53.1	1.75	290	33.3	1.64	1.1 (0.9–1.4)	1.2 (0.9–1.5)	71	7.5	1.07	0.7 (0.5–1.1)	0.9 (0.6–1.4)	53	6.2	0.89	**0.6** **(0.4–0.9)**	0.9 (0.6–1.4)
3rd quartile	605	59.0	1.76	322	32.0	1.61	1.0 (0.8–1.2)	1.1 (0.8–1.4)	46	4.4	0.70	**0.4** **(0.3–0.6)**	0.6 (0.4–1.0)	42	4.6	0.84	**0.4** **(0.2–0.7)**	0.7 (0.4–1.2)
4th quartile	532	55.5	1.80	332	36.8	1.83	1.1 (0.8–1.4)	1.2 (0.9–1.6)	43	4.7	0.72	**0.5** **(0.3–0.8)**	0.7 (0.4–1.3)	32	3.1	0.55	**0.3** **(0.2–0.5)**	0.5 (0.3–1.0)
**Current use of cigarettes**																		
Someday use	458	49.0	1.84	199	23.1	1.65	ref	ref	129	14.6	1.36	ref	ref	119	13.3	1.31	ref	ref
Daily use	1737	56.1	1.09	1049	35.2	1.04	**1.3** **(1.0–1.7)**	1.0 (0.8–1.4)	132	4.4	0.42	**0.3** **(0.2–0.4)**	**0.4** **(0.2–0.5)**	123	4.2	0.43	**0.3** **(0.2–0.4)**	**0.3** **(0.2–0.5)**
**Current use of ENDS**																		
No use	1053	54.1	1.34	595	31.8	1.24	ref	ref	138	7.3	0.72	ref	ref	133	6.7	0.71	ref	ref
Someday use	942	53.0	1.43	596	35.8	1.26	1.0 (0.8–1.1)	0.8 (0.6–1.1)	84	5.0	0.59	**0.7** **(0.5–1.0)**	0.8 (0.5–1.2)	97	6.2	0.64	0.8 (0.5–1.1)	1.0 (0.6–1.7)
Daily use	197	65.3	2.98	54	18.2	2.66	**0.4** **(0.3–0.6)**	**0.3** **(0.2–0.5)**	39	12.6	2.06	1.3 (0.8–2.2)	1.3 (0.7–2.2)	12	3.9	1.06	**0.4** **(0.2–0.8)**	0.5 (0.2–1.0)
**Current use of traditional cigars/cigarillos/filtered cigars**																		
No use	941	49.4	1.42	680	36.9	1.34	ref	ref	124	6.9	0.69	ref	ref	120	6.8	0.63	ref	ref
Someday use	1047	59.5	1.38	476	28.9	1.22	**0.7** **(0.6–0.9)**	0.7 (0.5–1.0)	106	6.0	0.66	**0.7** **(0.5–1.0)**	0.7 (0.4–1.2)	98	5.7	0.64	0.8 (0.6–1.1)	1.0 (0.6–1.7)
Daily use	170	62.2	3.23	58	20.5	2.47	**0.4** **(0.3–0.6)**	**0.3** **(0.2–0.5)**	26	10.5	2.30	1.4 (0.8–2.6)	1.6 (0.8–3.5)	20	6.7	1.52	1.0 (0.6–1.8)	1.2 (0.5–2.6)
**Current use of pipe**																		
No use	1993	53.7	0.97	1182	33.3	0.95	ref	ref	237	6.5	0.50	ref	ref	230	6.4	0.48	ref	ref
Someday use	193	64.9	3.13	61	21.8	2.98	**0.7** **(0.5–1.0)**	1.0 (0.6–1.6)	22	8.6	2.28	1.1 (0.6–2.1)	1.5 (0.8–2.7)	11	4.6	1.53	0.8 (0.4–1.7)	1.1 (0.4–2.9)
Daily use	6	41.9	12.85	4	34.0	14.13	1.7 (0.4–6.8)	1.4 (0.2–10.9)	2	17.7	10.33	3.5 (0.6–19.6)	3.2 (0.5–20.1)	1	6.4	7.72	2.0 (0.1–41.3)	2.3 (0.1–68.7)
**Current use of hookah**																		
No use	1590	53.7	0.96	991	34.3	0.94	ref	ref	180	6.4	0.56	ref	ref	156	5.6	0.52	ref	ref
Someday use	590	57.5	2.02	254	26.5	1.81	0.9 (0.7–1.1)	1.0 (0.7–1.4)	79	7.6	0.83	1.1 (0.8–1.6)	1.1 (0.6–1.8)	82	8.3	1.05	1.1 (0.7–1.7)	1.4 (0.8–2.5)
Daily use	14	54.2	13.75	1	8.8	9.20	0.3 (0.0–4.7)	0.6 (0.0–7.6)	2	17.6	11.99	3.3 (0.4–26.4)	4.2 (0.5–34.8)	3	19.4	11.39	3.0 (0.5–17.8)	5.1 (0.4–62.6)
**Current use of smokeless/** **snus pouches/ dissolvable**																		
No use	1674	51.2	1.05	1125	36.0	1.00	ref	ref	189	6.0	0.52	ref	ref	211	6.8	0.54	ref	ref
Someday use	345	68.7	2.28	99	21.9	1.99	**0.5** **(0.4–0.7)**	**0.6** **(0.4–0.8)**	27	5.2	1.44	0.6 (0.3–1.1)	0.6 (0.3–1.3)	20	4.2	0.94	0.6 (0.3–1.1)	0.8 (0.4–1.6)
Daily use	142	71.6	3.54	7	4.0	1.37	**0.1** **(0.0–0.2)**	0.1 (0.0–0.1)	39	20.2	3.25	**2.1** **(1.2–3.7)**	1.8 (0.9–3.3)	8	4.2	1.72	0.6 (0.2–1.8)	0.6 (0.2–2.0)

ⱡ Table 4 notes. Sample includes adults aged 18+ years who were ‘poly cigarette’ smokers at W1 and also participated in W2 (N = 3946); analyses weighted using W2 longitudinal weights. Results from multinomial logistic regression analyses indicate the relative probability of being in each W2 transition group vs the W2 ‘no transition’ group as a function of correlates assessed at W1. Current use at each wave defined for cigarettes as having smoked at least 100 cigarettes in lifetime and now smoking everyday or somedays; for hookah as now using everyday, somedays, or monthly; for all other products as now using everyday or somedays. ‘Poly cigarette’ use defined as current use of cigarettes and current use of at least one other tobacco product. ‘Exclusive cigarette’ use defined as current use of cigarettes and no current use of any other tobacco product. ‘Non-cigarette tobacco’ use defined as no current use of cigarettes and current use of any other tobacco product. Bold font indicates significant association at α = 0.05; grey shaded cells indicate unstable estimate due to denominator <50 or relative standard of the estimate >30%. ^1^ Analyses adjusted for age, sex, sexual orientation, race/ethnicity, education, and poverty level. ^2^ Analyses adjusted for age, sex, sexual orientation, race/ethnicity, education, poverty level, total number of products used, nicotine dependence, and type and frequency of product used. ^3^ Mean cumulative nicotine dependence score; quartiles determined among W1 ‘poly cigarette’ smokers. ^a^ No transition is defined relative to ‘poly cigarette’ use; therefore, no transition may reflect the same or different combinations of products used at W2 as were used at W1.
